# Zinc and copper levels are not correlated with angiographically-defined coronary artery disease in sudanese patients

**DOI:** 10.3389/fphys.2015.00191

**Published:** 2015-07-06

**Authors:** Mohamed F. Lutfi, Ramaze F. Elhakeem, Raga S. Khogaly, Abdelkarim A. Abdrabo, Ahmed B. Ali, Gasim I. Gasim, Ishag Adam

**Affiliations:** ^1^Faculty of Medicine, Alneelain UniversityKhartoum, Sudan; ^2^Faculty of Laboratory Science, Sudan International UniversityKhartoum, Sudan; ^3^Faculty of Laboratory Science, Alneelain UniversityKhartoum, Sudan; ^4^Faculty of Medicine, University of KhartoumKhartoum, Sudan

**Keywords:** angiography, angina, coronary artery, copper, zinc

## Abstract

We investigated zinc and copper levels in angiographically defined obstructive coronary artery disease (CAD) in patients undergoing elective coronary angiography in El-Shaab Hospital, Sudan. We performed a cross-sectional study. One hundred forty-two patients were enrolled. Sociodemographic and medical characteristics were collected using a questionnaire. Glucose, lipid, zinc, and copper levels were measured. Out of 142 patients, 102 (71.8%) had CAD and 40 (28.2%) had patent coronary arteries. There were no significant differences in median (interquartile range) zinc [118.5 (97.2–151.0) vs. 130.0 (106.0–174.0) μg/ml, *P* = 0.120] and copper [150.6 (125.0–183.0) vs. 158 (132.0–180.0) μg/mL, *P* = 0.478] levels between patients with CAD and those with patent coronary arteries. In linear regression analysis, there were no associations between CAD and zinc and copper levels. The current study failed to show any significant association between CAD and zinc and copper levels.

## Introduction

Coronary artery disease (CAD) is a large health problem worldwide and one of the leading causes of morbidity and mortality (Yusoff, 2002). Traditional risk factors of CAD, such as hypercholesterolemia, hypertension, and smoking, account for ≤50% of mortality (Gey et al., 1991). Therefore, other factors that might be involved in the pathogenesis of CAD need to be determined. There is growing evidence that oxidative free radicals play a role in the pathogenesis of CAD (Ames et al., 1993). Similarly, trace elements (e.g., zinc and copper) have also been implicated in the pathogenesis of CAD (Lukaski et al., 1988; Reunanen et al., 1996; Cebi et al., 2011). Research on zinc and copper in patients with CAD is important because these trace elements may be useful predictors for CAD and their levels might be able to be adjusted to prevent this disease. We have previously studied zinc and copper in different diseases in Sudan (Abdelrahim et al., 2009; Bushra et al., 2010; Mohamed et al., 2011); however, levels of these elements were not explored in Sudanese patients with CAD.

In Sudan, CAD is a large health problem. Coronary catheterization centers are mostly confined to the capital, Khartoum state. There are no published data on trace elements among Sudanese patients with CAD (Suliman, 2011; Musa et al., 2013). The current study aimed to investigate zinc and copper levels in patients undergoing elective coronary angiography because of typical attacks of angina.

## Materials and methods

We performed a cross-sectional study at the Cardiac Catheterization Center in El-Shaab hospital, Sudan, from November 2012 to May 2013. The patients were visited on the intended day of the procedure before performing cardiac catheterization. Following signed informed consent, sociodemographic characteristics, and the medical history (gender, age, smoking, hypertension, and diabetes mellitus) were assessed using a questionnaire. Blood pressure was measured using a mercury sphygmomanometer and mean arterial blood pressure was determined as diastolic blood pressure + [(systolic blood pressure–diastolic blood pressure)/3]. Weight and height were measured and body mass index (BMI) was calculated using the following formula: BMI (kg/m^2^) = weight (kg)/(height (m^2^). Fasting blood glucose levels and the lipid profile were measured using enzymatic methods. A 3 ml volume of blood was taken and allowed to clot. The sample was then centrifuged at 2500 rpm for 15 min at room temperature to obtain serum. The serum was stored at −20°C and used to measure zinc and copper by atomic absorption spectrophotometry (SOLAAR, Atomic Absorption Spectrophotometer; Thermo Electron, Cambridge, UK), as previously described (Ilyas et al., 2015). Coronary angiograms were performed by one of the authors (ABA) who is a cardiologist. The findings of coronary angiograms were interpreted as follows: the presence of one or more stenoses in ≥ half of the diameter of at least one major coronary artery (left main, right coronary, left anterior descending, and circumflex arteries) was considered as CAD (Marroquin et al., 2004).

### Data analysis

Data were entered into a computer using SPSS for windows version 19.0 (SPSS Inc., Chicago, IL, USA). Continuous data are described as mean (SD) when normally distributed and median (25–75% interquartile range) when not normally distributed. Zinc and copper levels were not normally distributed and the Mann–Whitney U test was used to assess significant differences between the two groups. Linear regression analysis was performed with zinc and copper levels as continuous dependent variables. Sociodemographic and biochemical characteristics were the independent variables of interest. *P* < 0.05 was considered significant.

### Ethics

The study received ethical clearance from the ethics review committee at the Faculty of Medicine, University of Khartoum, Sudan.

## Results

There were 142 patients enrolled in the study. Their mean (SD) age was 57.7 (13.1) years. The majority of the patients were males (94, 77.5%). Of these 142 patients, 68 (48.2%) were hypertensive, 55 (39.0%) had diabetes, and 18 (12.8%) had both of these conditions.

**Figure 1:**
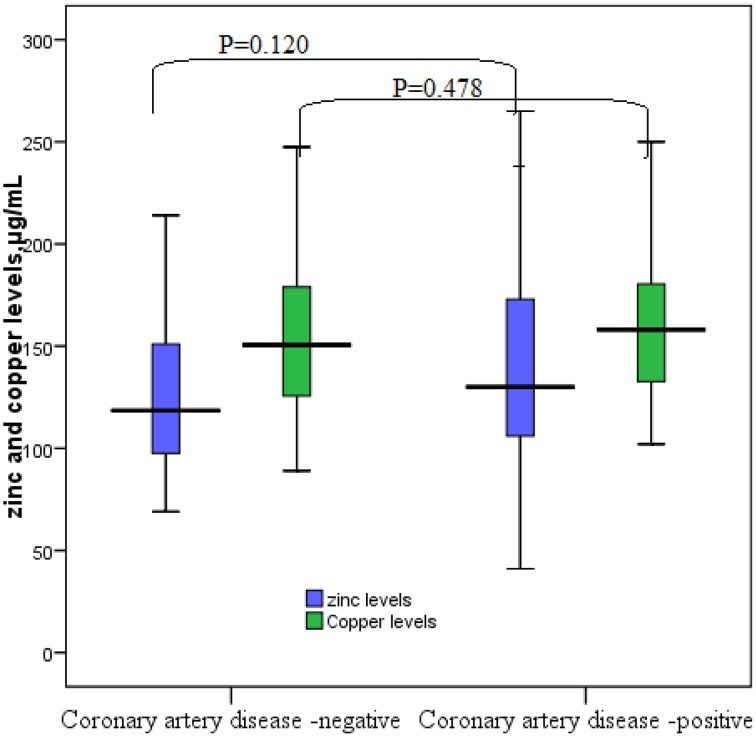
**Zinc and copper levels in patients with coronary artery disease**.

**Figure 2:**
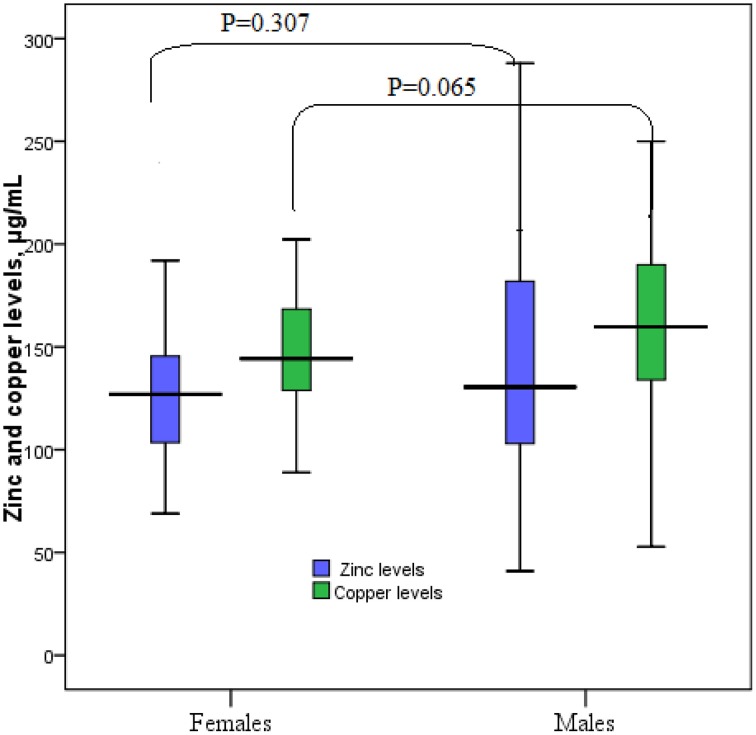
**Zinc and copper levels in males and females**.

The general medical and biochemical characteristics of these 142 patients are shown in Table 1. The median (interquartile range) of zinc and copper levels was 128 μg/ml (103–162 μg/ml) and 156 μg/ml (131–180 μg/ml), respectively. Coronary catheterization showed that 101 (71.8%) patients had CAD and 40 (28.2%) patients had patent coronary arteries. Median zinc and copper levels were not significantly different between women and men, obese (BMI > 30.0 kg/m^2^) and non-obese patients, hypertensive and non-hypertensive patients, and diabetic and non-diabetic patients. Similarly, there were no significant difference in median zinc and copper levels in patients with CAD and in those with patent coronary arteries (Table 2, Figures 1, 2).

**Table 1:** **Clinical and biochemical characteristics of the patients**.

**Variables**	**Mean (SD)**
Mean arterial blood pressure, mmHg	96.4 (12.2)
BMI, kg/m^2^	27.2 (4.9)
Hemoglobin, g/dl	13.3 (1.4)
Creatinine, mg/dl	1.8 (0.9)
White blood cells, cells /mm^3^	6.7251 (2.505)
Random blood sugar, mg/dl	155.4 (75.7)
Triglycerides, mg/dl	107.1 (58.4)
Cholesterol, mg/dl	150.4 (41.4)
LDL, mg/dl	86.6 (31.1)
HDL, mg/dl	39.0 (9.0)

**Table 2:** **Zinc and copper levels in the different groups of patients**.

**Variables**		***n*(%)**	**Zinc μg/mL**	**Copper μg/mL**
Gender	Male	94 (66.7)	130.0 (102.7–182.2)	159.7 (133.6–190.5)
	Female	47 (33.3)	127.0 (103–146)	144.0 (127.0–168.0)
*P*			0.307	0.065
Smoking	Yes	69 (48.9)	129.0 (105.0–176.0)	161.0 (134.0–187.0)
	No	72 (51.1)	127.0 (102.0–158.5)	149.0 (130.0–178.5)
*P*			0.840	0.324
Obesity	Yes	43 (30.5)	118.5 (102.7–136.0)	147.5 (130.4–169.7)
	No	98 (69.5)	132.0 (102.0–176.6)	159.0 (131.0–189.0)
*P*			0.116	0.179
CAD	Positive	40 (28.4)	118.0 (97.2–151.0)	150.6 (125.0–183.0)
	Negative	101 (71.6)	130.5 (106.0–174.0)	158 (132.0–180.0)
*P*			0.120	0.478

Linear regression analysis showed that none of the investigated factors were significantly associated with zinc levels. However, fasting blood sugar levels (*r* = −0.131, *P* = 0.011) and zinc levels were significantly associated with copper levels (Table 3).

**Table 3:** **Linear regression analysis of factors associated with zinc and copper levels**.

**Variable**	**Zinc level**			**Copper level**		
	**Coefficient**	***SE***	***P***	**Coefficient**	***SE***	***P***
Age	−0.239	0.487	0.625	−0.332	0.292	0.258
Gender	10.664	14.060	0.450	9.411	8.447	0.268
Mean arterial blood pressure, mmHg	−0.145	0.371	0.697	−0.017	0.222	0.938
Smoking	−7.198	10.620	0.499	3.515	6.377	0.583
BMI, kg/m^2^	−0.548	1.145	0.633	−0.543	0.687	0.431
Hemoglobin, g/dl	1.221	3.948	0.758	−2.832	2.366	0.234
Creatinine mg/dl	−31.218	18.954	0.103	3.606	11.508	0.755
White blood cells, cell × 10^3^/ml l	−0.003	0.002	0.141	0.001	0.001	0.519
Fasting blood sugar, mg/dl	0.030	0.084	0.727	−0.131	0.051	0.011
Triglycerides, mg/dl	−0.010	0.107	0.925	0.101	0.064	0.116
Cholesterol, mg/dl	−0.134	0.304	0.661	−0.173	0.183	0.344
LDL, mg/dl	0.418	0.332	0.212	0.146	0.201	0.468
HDL, mg/dl	−0.641	0.796	0.422	0.479	0.478	0.319
Coronary artery disease	15.421	13.704	0.263	3.875	8.262	0.640
Zinc	–	–	–	0.355	0.060	0.000

## Discussion

The main findings of the current study were that 28.2% of patients who underwent diagnostic cardiac catheterization had patent coronary arteries. In addition, serum zinc and copper levels were not different between CAD-negative and CAD-positive patients. Previous reports have shown that 10–40% of patients with anginal chest pain have normal coronary angiograms (Vermeltfoort et al., 2010; Douglas et al., 2011).

Recently, Murr et al. reported that zinc levels were not different between patients with CAD and controls (Murr et al., 2012). Interestingly, a previous study showed that although serum zinc levels were not associated with the prevalence/severity of CAD, urinary zinc loss was significantly higher in patients with CAD and was positively associated with the severity of CAD. Additionally, the serum zinc/24-h urine zinc ratio was inversely associated with CAD (Giannoglou et al., 2010). However, another study reported significantly lower serum levels of zinc and copper in CAD patients compared with those with a normal angiogram in 114 Persian patients who had a coronary catheterization (Kazemi-Bajestani et al., 2007). Later on, only a weak positive association between serum copper levels and calculated 10-year coronary risk was observed in a larger Persian cohort (Ghayour-Mobarhan et al., 2009). Although this study with a larger cohort failed to show a significant difference in serum zinc levels between the studied groups, the serum zinc/copper ratio was strongly inversely associated with the 10-year coronary risk. There is growing evidence that serum copper levels, but not zinc levels, are positively correlated with serum levels of leptin, which can control some CAD risk factors, such as body weight and the serum lipid profile (Olusi et al., 2003; Mohammadzadeh and Zarghami, 2013). Generally, low serum zinc levels are associated with an increased prevalence of CAD as the rest of CAD risk factors, such as diabetes, hypertension, and hypertriglyceridemia. Increased serum copper levels have been observed in patients with CAD (Roth and Kirchgessner, 1981; Reunanen et al., 1996; Singh et al., 1998). Similarly, Lukaski et al. observed a slight increase in serum copper levels, but a significant increase in urine copper levels in patients with myocardial infarction (Lukaski et al., 1988). Copper plays a pivotal role in atherogenesis, possibly through its catalyst effects of low-density lipoprotein oxidation and activation of several cholesterogenic genes in macrophages (Esterbaur et al., 1992; Svensson et al., 2003).

An imbalance in the metabolism of zinc and copper has been suggested to predispose to CAD. Therefore, a diet that is deficient in copper *per se* or relatively deficient on comparing to zinc, can lead to hypercholesterolemia and an increased susceptibility to atherosclerosis (Klevay, 1975). However, a large, cross-sectional study (600 individuals) showed no significant associations between serum zinc, copper, or the zinc/copper ratio and total serum cholesterol, high-density lipoprotein, low-density lipoprotein, or triglyceride levels (Neggers et al., 2001).

Limitations of the current study are that neither total zinc nor iron levels were measured and the dietary intake was not calculated. Another limitation is the absence of an angina-free control group for comparing between normal individuals and those with typical angina but with a normal angiogram.

## Conclusions

This small sample study shows that serum zinc and copper levels cannot predict eventful coronary angiography if patients experience typical anginal attacks. Further larger studies (e.g., longitudinal studies) are required.

### Conflict of interest statement

The authors declare that the research was conducted in the absence of any commercial or financial relationships that could be construed as a potential conflict of interest.
